# Profile of patients undergoing treatment with long-acting injectable antipsychotic drugs in a psychiatry hospital in Portugal

**DOI:** 10.1192/j.eurpsy.2023.336

**Published:** 2023-07-19

**Authors:** J. R. P. Correia, R. A. Moreira, E. Maldonado, H. J. Gomes, J. R. Gomes

**Affiliations:** Departamento de Psiquiatria e Saúde Mental, Unidade Local de Saúde do Nordeste, Bragança, Portugal

## Abstract

**Introduction:**

The treatment with long-acting injectable antipsychotics (LAIAs) is more and more frequent and it shows advantages regarding adherence, effectiveness and tolerance.

**Objectives:**

To describe and compare the profile of patients under treatment with LAIAs in a psychiatric hospital in Portugal.

**Methods:**

An observational and retrospective study was carried out with the collection of data referring to patients hospitalized with a first psychotic episode between 01/01/2019 and 30/06/2022 in a psychiatric hospital in Portugal and the respective evaluation of sociodemographic and clinical data through the information recorded in the clinical files.

**Results:**

During the 42 months of the study, we selected 78 patients who presented psychotic symptoms on admission. Patients with a history of previous psychotic episodes and prescription of antipsychotic therapy prior to hospitalization were excluded.

Of 78 patients hospitalized with a first psychotic episode, 34 - which corresponds to approximately 44% - were discharged with LAIAs.

Patients receiving LAIAs had an average age of 39 years. The average number of days of hospitalization was 28 days; 41% were female (n=14) and 59 were male (n=20); 35% (n=12) consumed psychoactive substances previously on admission to hospital; 62% (n=21) were discharged under the compulsive treatment regimen. Regarding the diagnosis at discharge, based on the international classification of disease-11 (ICD-11), the most common were schizophrenia, psychotic disorder induced by psychoactive substances and acute and transient psychotic disorder.

From the statistical analysis carried out, no correlation was observed between the rate of readmissions and the administration of LAIAs, nor was there any correlation between the rate of readmissions and compulsive outpatient treatment.

**Conclusions:**

Despite what is described in the literature, in the sample under study, the LAIAs were not superior in the variables studied, namely in reducing the readmission rate. Possible explanations for the results obtained may be justified by the size of the sample under study and the follow-up time of the cases.

**Disclosure of Interest:**

None Declared

